# Barriers and facilitators to medical care retention for pediatric systemic lupus erythematosus in South Africa: a qualitative study

**DOI:** 10.21203/rs.3.rs-3919073/v1

**Published:** 2024-02-28

**Authors:** Naira Ikram, Laura B Lewandowski, Melissa H Watt, Christiaan Scott

**Affiliations:** 1. Harvard Medical School, Boston, MA, 02115; 2. National Institute of Arthritis, Musculoskeletal, and Skin Diseases, NIH, DHHS, 9000 Rockville Pike, Building 10, 12N248 Room 28, Bethesda, MD 20892-1102, USA; 3. Duke Global Health Institute, 310 Trent Drive, Duke University, Durham, NC, 27710, USA; 4. University of Utah, Utah, USA; 5. Red Cross War Memorial Children’s Hospital, University of Cape Town, Klipfontein Road, Rondebosch, Cape Town, Western Cape, South Africa

**Keywords:** Africa, lupus, pediatric lupus, chronic disease, qualitative, South Africa

## Abstract

**Background:**

Systemic lupus erythematosus (SLE) is a life-threatening, chronic, autoimmune disease requiring long term subspecialty care due to its complex and chronic nature. Childhood-onset SLE (cSLE) is more severe than adult-onset, and the cSLE population in South Africa has been reported to have an even higher risk than patients elsewhere. Therefore, it is critical to promptly diagnose, treat, and manage cSLE. In this paper, we aim to describe and evaluate barriers and enablers of appropriate long-term care of cSLE South Africa from the perspective of caregivers (parents or family members).

**Methods:**

Caregivers (n=22) were recruited through pediatric and adult rheumatology clinics. Individuals were eligible if they cared for youth (≤19 years) who were diagnosed with cSLE and satisfied at least four of the eleven ACR SLE classification criteria.

Individual in-depth, semi-structured interviews were conducted between January 2014 and December 2014, and explored barriers to and facilitators of ongoing chronic care for cSLE. Data were analyzed using applied thematic analysis.

**Results:**

Four barriers to chronic care engagement and retention were identified: knowledge gap, financial burdens, social stigma of SLE, and complexity of the South African medical system. Additionally, we found three facilitators: patient and caregiver education, robust support system for the caregiver, and financial support for the caregiver and patient.

**Conclusion:**

These findings highlight multiple, intersecting barriers to routine longitudinal care for cSLE in South Africa and suggest there might be a group of diagnosed children who don’t receive follow-up care and are subject to attrition. cSLE requires ongoing treatment and care; thus, the different barriers may interact and compound over time with each follow-up visit. South African cSLE patients are at high risk for poor outcomes. South African care teams should work to overcome these barriers and place attention on the facilitators to improve care retention for these patients and create a model for other less resourced settings.

## Background

Systemic lupus erythematosus (SLE) is a life-threatening, chronic, autoimmune disease that manifests through multisystem episodic inflammation and causes severe morbidity and early mortality. Accordingly, SLE requires long term subspecialty care due to its complex and chronic nature (1, 2). Worldwide, rheumatic diseases like SLE are understudied despite being the second leading cause of disability adjusted life years, as measured by years lost to disability (3). Genetic predisposition coupled with healthcare disparities in medically underserved areas exacerbate SLE outcomes (4). In high-income countries, individuals of African descent experience SLE at a higher rate and have more severe symptoms and worse outcomes (5). Childhood-onset SLE (cSLE) is more severe than adult-onset SLE, as children have higher disease activity, require more medications, and accrue more damage than adult counterparts (6). Therefore, it is critical to promptly diagnose, treat, and manage cSLE. cSLE is a chronic disease with no cure and requires routine follow-up visits for disease monitoring and management. Managing cSLE requires a multidisciplinary team to oversee clinical management in order to educate families about prevention of disease flares which can lead to irreversible damage, address concerns about reproductive health, encourage medication adherence, acknowledge the mental health burden of chronic illness, and ease the transition to medical care upon adulthood (6). Early recognition and ongoing access to care and care retention impact outcomes, preventing disease progression and complications (6).

In a recent study with participation from 44 African countries, 12 countries have between one and ten rheumatologists while 17 have no rheumatologists; the majority of rheumatologists are in Northern Africa (7). South Africa is a lower-middle income country that is a leader in health care for Africa, yet the health system still has significant challenges and disparities. Despite the increasing burden of chronic diseases in South Africa, little is known about the barriers to accessing and utilizing health care for pediatric chronic conditions. South Africa is burdened with poverty, communicable diseases such as tuberculosis and human immunodeficiency virus (HIV), racial tension, community violence and malnutrition (8). South Africa is still dealing with repercussions of its apartheid history and subsequent entrenched far-reaching disparities (9). Ongoing health care delivery to patients with chronic disease requires coordination of most aspects of the medical system: health financing, governance, health workforce, health information, medical products and technologies, and health-service delivery (10).

In South Africa, cSLE is characterized by patients who have more active disease, serious organ system involvement, and worse outcomes than North American peers (11, 12). Previously, our team demonstrated that there are many barriers to diagnosing cSLE in South Africa. Among patients currently receiving care for cSLE, they face challenges at the level of cSLE caregivers (lack of knowledge about SLE, financial difficulties, social stigma of SLE) and the health system (misdiagnosis, lack of trained staff, a complex medicine systems) (13). Continued long term medical care is essential for cSLE maintenance, as lapses in care are linked to disease flares (14). The barriers to receiving sustained medical care in this population have not been explored, and it is not known how they differ from the barriers experienced in receiving an initial diagnosis. The purpose of this study is to describe caregivers’ experiences engaging in long term medical care for cSLE, and to identify barriers and facilitators to long term care retention among cSLE patients. This study utilizes a qualitative approach to understand the experience of managing cSLE in the context of the South African healthcare system.

## Methods

### Setting

Participants were recruited through pediatric and adult rheumatology clinics at two government-funded tertiary hospitals (Red Cross War Memorial Children’s Hospital and Groote Schuur Hospital) and a private practice clinic in Cape Town, South Africa.

### Participant Recruitment

Family caregivers were considered eligible for recruitment if they cared for youth (<19 years) who were diagnosed with cSLE and satisfied at least four of the eleven ACR SLE classification criteria. Out of the potential caregivers for the 72 patients identified across the three centers, we attempted to contact each family unit to identify a named contact for study participation. Thirty were contacted successfully in-person, by phone, or by email. Of the 30 contacted, 22 cSLE caregivers enrolled in the study and completed an interview.

### Ethical review

The study was approved by the Duke Institutional Review Board (#Pro00045133) and University of Cape Town Human Research Ethics Committee (#424/2013). Participants completed written informed consent in their preferred language prior to the interview.

### Procedures

Individual, in-depth, semi-structured interviews were conducted between January 2014 and December 2014. The interview guide was developed based on a literature review on specific hurdles embedded in the South African medical system and challenges in managing cSLE. The guide included open-ended questions and follow-up probes about the barriers to and facilitators of ongoing long-term care for cSLE. Participants were offered the flexibility of having the interview conducted at home (n = 3) or at the clinic attended (n = 19) to minimize the burden of travel. Interviews were conducted by LBL and lasted 30 to 90 minutes. Interviews were conducted in the primary language of participants (English [n = 16]; Afrikaans [n = 3]; Xhosa [n = 3]). Non-English interviews required a linguistically and culturally competent translator who provided real-time, simultaneous translation.

Self-described participants were requested to report how they self-identify (racially/ethnically) with categories based on those in South African population surveys (15).

### Analysis

The interviews were audio-recorded and transcribed verbatim. They were analyzed using applied thematic analysis to identify recurring themes related to barriers to and facilitators of chronic care management perceived by caregivers of cSLE patients (16). Major themes were reviewed by two coders to reach consensus, and analytic memos were written to organize the content of the interviews and incorporate relevant quotes. The memos were manually coded for primary thematic categories related to chronic care retention barriers and facilitators at the levels of the patient and/or health system.

## Results

There were 22 cSLE caregivers in the sample: 18 mothers, 2 fathers, 1 grandmother, and 1 foster mother. In all cases, the race/ethnicity of the caregiver was consistent with that of the child. Descriptive characteristics, such as age, household income, means of transportation, and education level, are described in Table 1. Although 36% of South Africans live below the poverty line (R620/$43 USD per month), none of the caregivers reported monthly household incomes below this threshold (17, 18).The majority of caregivers (59%) received government social welfare grants (R450/$29 USD per month), and would likely live below the poverty line without the grants.(19) Eighteen percent of caregivers reported living without running water or electricity.

### Barriers

Four barriers to engagement and retention in cSLE were identified (Table 2). Three barriers were at the level of the caregiver (knowledge gap, financial burdens, and social stigma of SLE) and one barrier was at the level of the health system (complexity of the South African medical system).

### Caregiver Knowledge Gap

Most caregivers expressed little to no prior knowledge of SLE. Subsequently, caregivers were unable to distinguish SLE symptoms from other illnesses, and severe symptoms from typical ones. Upon receiving the diagnosis of cSLE, many caregivers still reported confusion over diagnosis and were unable to comprehend the disease itself, much less be able to define it for others. One caregiver conveyed that “[if someone asks what lupus is], I just only told them when she had the first bad problem, then I couldn’t explain it. I can’t really explain it.” In addition to the lack of understanding of cSLE at diagnosis, there was a knowledge gap in chronic care management of SLE. For example, many caregivers did not understand that the medications for SLE are not only to be used improve symptoms during a flare, but also to maintain disease control and prevent flare, and need to be taken even when the patient feels well. Additionally, many caregivers did not realize that severe damage that occurs prior to proper treatment cannot always be reversed, even with optimal therapeutics.

Furthermore, the caregivers did not always envision themselves as partners of the healthcare team in managing cSLE over time. Patient-centered care and shared decision making are increasingly recognized as pillars of quality healthcare. Shared decision making can empower patients and families to take an active role in their care (20). There has been a slower adoption of these practices in LMIC settings (21). Yet, there is a hidden burden on caregivers to manage cSLE, transferring most of the effort from the health system and providers to caregivers who lack experience and resources.

### Financial Burden of Care

The financial burden of cSLE longitudinal management was the most frequently cited issue. There is an economic burden associated with a chronic illness like SLE. The burden includes specific treatment costs, transportation, and caregiver loss of productivity/income (22). Caregivers who were above the poverty line pay medical bills for hospitalization on an income-based level, and many were on a payment plan for bills from initial diagnosis. Accessing sick leave for chronic illness management for their child was identified as a barrier by many caregivers. Although missing work once for the initial illness was viewed by employers as justifiable, missed work accumulated with repeated follow-up visits. Most caregivers reported insufficient sick leave to cover appointments at 2-to-3-month intervals. Some caregivers reported disappointment that their supervisors didn’t understand the critical timing of follow-up visits and prioritizing their child’s misunderstood health issues over work. Transportation costs were also a financial burden, as the majority of caregivers did not have access to private transportation and had to rely on public transportation. They had to pay to get to and from the medical facility, and the high transportation costs accumulated over time and were a burden on tenuous family finances. The time it took to commute to the clinic was great and costly. One caregiver stated that “we come to clinic every two weeks in a taxi…it takes 1 hour…last week [we didn’t have taxi fare], so we walked. [The walk] took three hours.” The time taken away from work at the visit itself, as well as the transportation to arrive to the clinic, are compounded by transportation costs from previous follow-up cSLE visits and ones for other health reasons and bills from initial hospitalization.

### Social Stigma of cSLE

While caregivers themselves had limited knowledge of cSLE, the caregivers reported that the general public appears to hold negative misconceptions about SLE. Caregivers feel the effects of this stigma, reporting that people outside their family unit, such as neighbors and school community members, believe that cSLE is contagious, when in reality “it’s only in [one’s] joints…[and] not contagious for somebody else to get.” Many caregivers cited that they felt like they had to hide their child’s condition from the community out of fear of lasting social stigmatization. Such stigma is a barrier to longitudinal care. Taking a child out of school for care may call attention to the cSLE diagnosis. If caregivers feel stigma about the illness, they are motivated to reduce actions which make the diagnosis more visible.

### Complexity of Medical System

Complexity of the medical system emerged as a barrier to continuing care. Sustained follow-up care is at the core of a chronic disease without a cure like cSLE yet required multiple visits just as difficult to navigate and access. cSLE patients often required consultation from additional specialists, such as nephrologists and hematologists, which made it even more difficult to coordinate appointments. The time and transportation costs noted above were multiplied if the family had to make a visit to two different specialists on different days of the week. The medical team did not always fully communicate the plan during disease maintenance and disease flares, which at times could lead to confusion.

### Facilitators

While barriers to continuing to manage care for SLE were identified in this study, themes around factors which greatly enabled chronic care emerged as well (Table 3). Three themes emerged for facilitators in chronic care of cSLE, all of which directly related to the caregiver level (patient and caregiver education, robust support system for the caregiver, and financial support for the caregiver and patient).

### Patient and Caregiver Education

Good communication and continued education by the pediatric rheumatologist were noted as facilitators to receiving ongoing care for cSLE. This was accomplished when the doctor thoroughly answered any questions and equipped the caregiver with informational tools. One caregiver expressed that “Dr. - was excellent…he could tell us what was wrong with [our daughter] …He gave us brochures and stuff as well.” Another said, “I think I’ve been well informed, and I ask questions every time.” The lack of caregiver knowledge on cSLE and chronic care management that was described as a barrier above had been reduced in some families through consistent and targeted education of caregivers. This education enabled caregivers to understand the importance of ongoing visits for chronic disease and helped them provide optimal care between appointments for their child. Caregivers also reported that their children with SLE also benefited when they were aware of their own condition and had enough background knowledge to form relevant questions.

### Social Support System for Caregiver

When caregivers were surrounded with a robust social support system, the barriers to caring for a child with cSLE were lessened. The support tended to come mainly from the parents of the caregiver and fulfilled every aspect of a solid support system, as one caregiver said that without “[having] to ask, [my parents] are always there for me.” Another caregiver was grateful that “my mother [gives me support.] If I can’t get off work, she will look after [my daughter when she is sick.].” In some cases, the caregiver’s employer was part of the support system if they provided work flexibility; as one parent summarized, her employer was “very understanding and supportive.”

### Financial Support for Caregiver and Patient

The financial burden of missing work (therefore going without pay) as well as the expenses involved in hospital bills and transportation costs that pile up with every follow-up visit are significant challenges. Support stems from “employers who have understood. So now when [my child] wasn’t feeling well I could stay at home and work from home. Also, when she was ill, I had a full-time job and they allowed me to stay out for the month she was ill.” Additional cited ways to lessen the financial burden on caregivers and patients extend beyond the workplace and into the realm of more convenient and less costly transportation to and from appointments. By providing financial support for the caregiver and the patient, this major barrier can be alleviated.

## Discussion

Childhood-onset SLE is a chronic condition, and consistent longitudinal care is necessary to keep the disease in remission and prevent long term damage. cSLE patients in South Africa have very active disease and poor long-term prognosis(11, 12), so it is critical to retain diagnosed patients in care to keep disease in remission and prevent inflammation and subsequent organ damage.

Our prior study highlighted missed opportunities for timely diagnosis of cSLE in South Africa, identifying the following barriers: knowledge gap regarding SLE, financial difficulties, social stigma of SLE, lack of trained staff, complex medical system, and misdiagnosis. In this study, similar themes related to barriers to care retention for South African cSLE patients arose. These findings imply that just as there might be a population of children who never receive diagnosis for cSLE, there might be a subset of diagnosed children who don’t receive follow-up care and are subject to attrition (13). Chronic diseases are defined by ongoing treatment and care; thus, the different barriers interact and compound over time with each follow-up visit. In contrast to acute management of illness, for which most care takes places in a medical setting, for chronic conditions the health system tends to place greater responsibility on caregivers rather than medical professionals (23, 24). Thus, chronic conditions place more demands on the physical capabilities, time, and emotional bandwidth of caregivers. In particular, the chronic nature of cSLE coupled with its multifaceted progression mandates that it is treated consistently and responsively, underlining the importance of the caregiver’s role in managing their child’s condition.

In the US, Canada, and Europe, access to care for chronic conditions is relatively easier to attain (25–27). In contrast, most low- and middle-income countries have historically prioritized infectious diseases and trauma, and have fewer medical professionals trained in managing pediatric rheumatic disease, as well as lack of access to proper testing and equipment for ongoing disease monitoring (23, 28). In fact, many infectious disease symptoms overlap with rheumatic disease such as inflammation and fever, making differentiation difficult especially when considering the contrasting treatment plans for these two categories of illness. South Africa is unique because it has built infrastructure for the increasing number of chronic conditions, partly due to the gradual introduction of AIDS care over the past few decades and shift from episodic treatment to life-long care (29). Some of the barriers described in this study likely extend to all areas of the world (appointment delays and a long wait to see a provider, gap in caregiver knowledge, social stigma, misdiagnosis), while others are specific to LMICs (transportation costs, complex medical system) (30).

The financial burden of travel and caregiver-missed work was identified as the greatest barrier to ongoing cSLE care in this setting. Most of the patient caregivers were receiving social grants which support inadequate household income, revealing some degree of financial instability. Continued missed work or use of sick leave could threaten employment, and subsequently the financial situation of the entire family. Most cSLE patients will need to travel to clinic for SLE management every one to three months. If an employer is unfamiliar with SLE or chronic disease management, caregivers may be penalized or terminated for continuing to miss working days. In a study of adult patients in South Africa, unskilled workers and domestic employees faced the largest barriers to sick leave for chronic disease management (31). Furthermore, if specific organ systems are affected by SLE, as is seen in lupus nephritis or central nervous system SLE, the patient may have more than one subspecialty visit to attend. Both hospital bills and the hidden costs of chronic care (taxi/transportation fare and missed work) compound at each follow-up visit, culminating in a greater burden on the caregiver. Many caregivers reported they are without access to running water or electricity and this suggests that patients with lower socioeconomic status can be vulnerable to opting out of ongoing care due to high cost (9). Structural racism contributes to adverse health outcomes through a variety of avenues, and racism is associated with poor physical health. The patients and caregivers in our cohort were primarily Black or of admixed ancestry, and therefore, likely affected by the impact of structural racism (32, 33). The majority of caregivers didn’t have access to private transportation, and subsequently had to rely on minibus taxis, which are public and the least expensive yet most dangerous mode of transportation in Cape Town (34). The financial barriers to cSLE care retention affect minority groups at a greater rate, partially explaining racial discrepancies in symptom severity and care received (5).

The complexity of the South African medical system was a barrier to timely SLE diagnosis and continues to impede ongoing care for SLE (10). In this study, the complexity of care was more dependent on multiple subspecialists involved in the care of cSLE. For some patients, they may see multiple providers from different subspecialties, which often hold clinic on different days. Therefore, the burden of missed work and cost of transportation to clinic is multiplied by number of subspecialty visits.

The lack of caregiver knowledge about cSLE is another barrier to continuing cSLE care. This knowledge barrier is not unique to SLE but is characteristic of many chronic diseases like diabetes and HIV/AIDS, which are widespread in South Africa (35, 36). In a study on diabetes in South Africa, the confusion over diabetes and its management is a reflection of poor access to healthcare, mistrust of that healthcare system, and/or little to no perceived social support (35). There are few pediatric rheumatology providers in sub-Saharan Africa, limited to major urban centers. Lack of understanding about disease, mechanism and prognosis may lead to misperception about the importance of ongoing clinic visits for disease control, and subsequent disease activity and damage (37). This is not unique to South Africa, because among healthcare workers in Kenya, there is varied understanding of the severity and subtypes of pediatric rheumatic disease leading to frustration among providers, patients, and their families (38). The limited knowledge of the diagnoses and management of chronic rheumatic diseases in general practitioners led to addressing the more urgent challenge cited was managing pain control rather than conclusively working towards diagnosis and treatment, which could lead to delays or lack of diagnosis.

cSLE is a relatively rare condition, and there is little public awareness which can lead to social stigmatization.(39) A disease typically becomes stigmatized if it is progressive and incurable, not well understood among the public, and presents symptoms that can’t be concealed (40). Often, there is stigma associated with a sick child (23). The stigma of a child with chronic disease may be more heightened than that of a chronically-ill adult, because the image disrupts expectations of a proper society with healthy children who are occasionally, if at all, susceptible to short-term illnesses and not ones conventionally viewed as byproducts of aging (41). Some of the symptoms of cSLE, like alopecia and malar rash, can’t be easily masked, and may instill fear in uninformed individuals of contagion and easy transmission (42). To protect their child from stigma, caregivers may isolate their family from a larger social network, which ultimately may result in lack of necessary support to continue to access care(43). Caregivers may attempt to conceal the signs of illness, which may include frequent visits to clinic or hospital. Lapsed visits may contribute to inadequate treatment and less contact with the pediatric rheumatologist may further deepen the knowledge barrier.

Perceived stigma can lead to self-imposed isolation and socially restricted lives by both patients and caregivers (23). Coping with this involves the difficult decision of a patient to either reveal his/her condition or conceal it and pass as normal (44). Children with cSLE are aware that they live with a life-long, unpredictable disease that is physically distinguishing and more severe than adult-onset SLE symptoms (1). On top of the multiple stressors imposed by society, patients are advised to limit certain behaviors and prioritize others, which might be perceived as restricting at times (42). Strained relationships often emerge, and patients sometimes react by noncompliance to treatments and nonadherence to medications (42, 45). The cumulative impact of social stigma is that cSLE patients with poor disease control are likely to have higher rates of depression, particularly those with lower education backgrounds (46). The mental health comorbidities of cSLE further impedes patients from optimally managing cSLE(47).

We identified three primary facilitators for cSLE patient retention: education, financial support, and development of a robust social support system for patients and caregivers (13). The possible solutions for each barrier are informed by other chronic disease models (24, 30, 35, 48). Creating robust, local support systems through caregiver networks and support groups may improve chronic care return rates for cSLE patients in South Africa. To reduce the complexity of chronic care and the added burden of multi-specialty care, patient navigators have been used in other health systems to improve care retention in SLE (49). Social and financial support are discussed together due to their interwoven, co-dependent nature. Financial support in the form of transportation, flexible shifts, and monetary contributions, for example, are extensions of social support and filling in the present needs of a household managing cSLE. In addition to financial systems to ease the burden of transportation costs, innovative solutions to ongoing care could be considered in this setting. The use of health tools such as patient education texts could improve patient and caregiver health literacy. Alternating in-person visits with phone or telehealth visits could reduce the burden of missed days on the family and improve the patient and caregiver connection. The recent global COVID-19 pandemic has increased the infrastructure for telehealth in SLE clinics, and this may have long-term benefits to reduce time and travel costs for some cSLE caregivers. (50–53). Adaptations for the virtual format are underway, such as the virtual pediatric gait, arms, legs, spine screening examination is a component of a global telemedicine initiative (54). Those families who lack access to internet services will continue to struggle with health care access.

Beyond direct financial support, support systems also include support groups, which have been proven to be effective on clinical and social outcomes, likely because peers might be perceived as more approachable and accessible than medical providers (30). Online support groups, such as WhatsApp group chats, are very promising in the current technology-driven world and can reduce the gap between clinical and at-home care (55). Similarly, other interventions that can be conducted by both medical and non-medical professionals, such as disease education and prevention programs, pain management strategies, school support, career planning, family counseling, and psychotherapy, can be broadly designed so that they are personalized for the patient for maximal benefit (46). The cumulative impact of support groups is the reduction of the knowledge gap in cSLE among caregivers, patients, and hopefully even extending to the public. Discussing and re-calibrating patient expectations should be the guiding force behind every intervention. Improving family education at diagnosis and follow-up visits may increase patient-physician trust, empower patients and caregivers, and motivate them to return for care.

This study has several limitations. Primarily, selection bias influenced the sample of caregivers, as those interviewed had been able to overcome barriers to attending follow-up visits. We were not able to capture the insights of caregivers for patients that had been diagnosed but lost to follow-up or those that were not diagnosed at all–these two subsets might have experienced different barriers which prevented them from coming back. Therefore, the barriers and facilitators described in this paper stem from a group of caregivers who have demonstrated resilience and had access to resources that enabled them to receive long-term subspecialized cSLE care. While some of the caregivers were below the poverty line, they received support via government social grants, which likely contributed to their successful retention. Additionally, the interviews were conducted in an urban setting at two government centers and one private practice, possibly creating selection bias. However, the likelihood of representative sampling was increased: 80% of South Africans use public health care (56), and the only cSLE providers in the region were located at these institutions.

Solutions to implement in the future should be grounded in the themes identified in the caregiver interviews (Tables 2 and 3). Since financial barriers were the most cited, transportation or transport subsidies arranged by employers would reduce the hassle of attending appointments, decreasing travel time, and thus reducing the amount of time away from work. A standardized letter written by the SLE care team to caregiver employers could be given to new patients and caregivers. The letter would explain the caregiver has a child/dependent with cSLE and the importance for follow-up on a schedule of 1–2 months. Such communication could minimize the unknown and confusing aspects of this disease and hopefully incite caregivers to seek routine care. SLE support groups would additionally improve caregiver and patient knowledge so they are empowered to return for care, and individuals that are part of their extended social network could be invited to attend, reducing social stigma and misconceptions surrounding cSLE. Clinic-based education sessions could complement caregiver or patient-led support groups to improve knowledge about SLE diagnosis and chronic management. Online support groups and educational materials are another format to be considered, although these may not be accessible for low-income families. Cohort monitoring could identify patients who have not returned to clinic, and patient- and family-centered interventions could be designed to optimize continued contact with the patient while minimizing the burden of travel and missed work on caregivers. Patient navigators are trained to help patients overcome modifiable barriers to health care and can improve care of chronic conditions. Navigators may be nurses, social workers, or lay health workers, including peers. Navigators trained in SLE may be a cost-effective way to retain families in care and reduce significant morbidity and mortality of cSLE.

## Conclusion

The management of a chronic yet life-threatening pediatric disease such as cSLE is a challenge worldwide. South African cSLE patients are at high risk for poor outcomes; therefore, identifying barriers and facilitators to ongoing care is important. This work highlights challenges to patients and caregivers for cSLE patients to continued care in clinic. South African care teams can work to implement changes, such as consolidating multidisciplinary care, creating virtual/WhatsApp patient support groups, and connecting families to social services to improve care retention for these patients and create a model for other, less resourced settings.

## Figures and Tables

**Table 1:** Demographic Characteristics of Pediatric SLE Sample (N = 22)

Characteristics	n	%
**Caregiver relation to patient**		
Mother	18	81
Father	2	9
Grandmother	1	5
Foster mother	1	5
**Self-reported race/ethnicity of caregiver**		
Admixed	16	73
Black	3	13
White	2	9
Indian	1	5
**Language of interview**		
English	16	72
Afrikaans	3	14
Xhosa	3	14
**Caregiver highest education level**		
Less than grade 12	8	36
Grade 12	8	36
University of higher	3	14
Not reported	3	14
**Access to private transportation**		
Yes	7	32
No	14	59
Unknown	2	9
**Household income, per month** *	Mean: R4,693 ($363 USD)	Range: R950 - R10,000 ($73–800 USD)
**Age of child SLE patient at diagnosis, years**	Mean: 10.9	Range: 7–15
**Age of caregiver, years**	Mean: 44.2	Range: 32–74

* R = South African Rand, USD = United states Dollars

**Table 2:** Barriers to long term SLE care retention: themes and illustrative quotations

Theme: Caregiver Knowledge Gap regarding SLE	
Caregiver, Age of child SLE diagnosis 12 yrs, Mean income R1200/ 93 USD*	“[If someone asks what lupus is], I just only told them when she had the first bad problem, then I couldn’t explain what it was. I can’t really explain it. “
Caregiver, Age of child SLE diagnosis 7 yrs, Mean income R1100 /85 USD	They only told me…it is just her kidneys…They told me the protein in the pee is too much and that is all they told me…so I didn’t know it was lupus.
Caregiver, Age of child SLE diagnosis 10 yrs, Mean income NR^†^	“I would just [say] ‘I am at the stage where I don’t explain lupus.’ Normally [I] tell them that, otherwise I would just say it is a disease that affects anyone at any age.”
Theme: Financial Barriers for Caregivers	
Caregiver, Age of child SLE diagnosis 10 yrs, Mean income NR	“We come to clinic every 2 weeks in a taxi…it takes 1 hour…last week [we didn’t have taxi fare], so we walked. [The walk] took three hours.”
Caregiver, Age of child SLE diagnosis 7 yrs, Mean income R1100 /85 USD	“It affects me…I will have people say come to a job, but I can’t because I am with my daughter. I can’t’ come every month because she is sick, sick leave is used up. But I must.”
Caregiver, Age of child SLE diagnosis 9 yrs, Mean income R6000 /465 USD	“After she was diagnosed, I got a job, but the boss was so nasty [about missed work], so I quit…the manager didn’t understand that [my daughter] had to come to the hospital…I applied for a grant, but I didn’t meet [eligibility].”
Caregiver, Age of child SLE diagnosis 10 yrs, Mean income NR	“It takes about an hour on the bus to get here. For me it is much safer with the bus than a taxi because a taxi is quite like they don’t care about your life, they just want to drive and get paid. That is why we take the bus, and it takes about an hour to come here.”
Caregiver, Age of child SLE diagnosis 9 yrs, Mean income R1500 /116 USD	“I got one bill for $2300 something and I was like what the hell, where am I going to get this money to pay this. I called the hospital and enquired about this, and they told me to go to the admin block and speak to people. I told them my husband is working years as a taxi driver and is only paid minimum.”
Theme: Social Stigma of sick child	
Caregiver, Age of child SLE diagnosis 13 yrs, Mean income R840 /67 USD	“I tell them that… it’s only in his joints and that it affects his joints and it’s not contagious for somebody else to get.”
Caregiver, Age of child SLE diagnosis 9 yrs, Mean income R6000 / 465 USD	“When people first know…it is like…they are scared, or something.scared they are going to get sick.”
Caregiver, Age of child SLE diagnosis 9 yrs, Mean income R1500/ 116 USD	“She was [bloated from prednisone] when she went to high school and then some children made fun of her and all.”
Caregiver, Age of child SLE diagnosis 10 yrs, Mean income NR	“I think there are a lot of misgivings about the whole Lupus spectrum; I don’t think many people are aware of it.”
Theme: Complexity of South African Medical System	
Caregiver, Age of child SLE diagnosis 12 yrs, Mean income R1200/ 93 USD	“My visits [for pediatric rheumatology care] take 3 hours, not including trips to the pharmacy. “
Caregiver, Child age of onset 10 yrs, Mean income NR	“[We have doctor visits for lupus] monthly and then after a certain time, maybe two months.Maybe after three months to six months and we have to come back to Cape Town. [from the Eastern Cape ~ 760 km/ 500miles]”

* R = South African Rand, USD = United States Dollars;

† NR = Not reported

**Table 3:** Facilitators to long term SLE care retention: themes and illustrative quotations

Theme: Patient/Parent Education	
Caregiver, Age of child SLE diagnosis 10 yrs, Mean income NR^†^	“I think I’ve been well informed, and I ask questions every time [I have a question.] I say ‘It’s your immune system. That your body is fighting itself.”
Caregiver, Age of child SLE diagnosis 11 yrs, Mean income R1625/ 125 USD*	“Dr. – was excellent…he could tell us what was wrong with {our daughter.] … He gave us brochures and stuff as well.”
Theme: Robust Caregiver support system	
Caregiver, Age of child SLE diagnosis 10 yrs, Mean income NR	“The people who give me the most support are my parents…we’re never alone if we need something, and I don’t even have to ask, they are always there for me.”
Caregiver, Age of child SLE diagnosis 10 yrs, Mean income NR	“My mother [gives me support.] If I can’t get off work, she will look after [my daughter when she is sick.] My employer: she’s been very understanding and supportive.”
Theme: Financial support	
Caregiver, Age of child SLE diagnosis 10 yrs, Mean income NR	“I’ve been lucky; actually, both of us have had employers who have understood. So now when she wasn’t feeling well I could stay at home and work from home. Also when she was ill, I had a full time job and they allowed me to stay out for the month she was ill.”
Caregiver, Age of child SLE diagnosis 10 yrs, Mean income NR	“[There were] definitely not [delays in diagnosis or treatment.] We have obviously been in a fortunate position to have private [medical care in the private health care system] treatment.”

* R = South African Rand, USD = United States Dollars;

† NR = Not reported

## Data Availability

The datasets generated during and analyzed during the current study are not publicly available, as the primary data consists of semi-structured interviews and contains information that could compromise the privacy of study participants. This data can be made available in part or full from the corresponding author (LBL) on reasonable request with proper privacy protection.

## Appendix

*The racial groups on the South African census are Black African, White, Admixed, Indian/Asian, or Other. (S.S. Africa Census 2011: Community Profile Databases Statistics South Africa, Pretoria (2012))

## Abbreviations

SLE: Systemic lupus erythematosus
cSLE: Childhood-onset SLE

## References

[1] Livingston B, Bonner A, Pope J. Differences in clinical manifestations between childhood-onset lupus and adult-onset lupus: a meta-analysis. Lupus. 2011;20(13):1345–55. 21951943 10.1177/0961203311416694

[2] Bilsborrow JB, Pelaez-Ballestas I, Pons-Estel B, Scott C, Tian X, Alarcon GS, Global Rheumatology Research: Frontiers, Challenges, and Opportunities. Arthritis & rheumatology (Hoboken, NJ). 2021. 10.1002/art.41980 PMC8712358 34535973

[3] Hoy D, March L, Brooks P Blyth F, Woolf A, Bain C, The global burden of low back pain: estimates from the Global Burden of Disease 2010 study. Annals of the Rheumatic Diseases. 2014;73(6):968–74. 24665116 10.1136/annrheumdis-2013-204428

[4] Fernandez M, Alarcon GS, Calvo-alen J, Andrade R, McGwin G Jr., Vila LM, A multiethnic, multicenter cohort of patients with systemic lupus erythematosus (SLE) as a model for the study of ethnic disparities in SLE. Arthritis care & research. 2007;57(4):576–84. 17471524 10.1002/art.22672

[5] Uribe AG, Alarcon GS. Ethnic disparities in patients with systemic lupus erythematosus. Current rheumatology reports. 2003;5(5):364–9. 12967518 10.1007/s11926-003-0022-8

[6] Ardoin SP Schanberg LE. The management of pediatric systemic lupus erythematosus. Nature Clinical Practice Rheumatology. 2005;1(2):82–92. 10.1038/ncprheum0046 16932637

[7] Dey D, Paruk F, Mody GM, Kalla AA, Adebajo A, Akpabio A, Women in rheumatology in Africa. Lancet Rheumatol. 2022;4(10):e657–e60. 38265961 10.1016/S2665-9913(22)00255-7

[8] Mayosi BM, Benatar SR. Health and Health Care in South Africa – 20 Years after Mandela. New England Journal of Medicine. 2014;371(14):1344–53. 25265493 10.1056/NEJMsr1405012

[9] Lewandowski LB, Scott C. Apartheid and healthcare access for paediatric systemic lupus erythematosus patients in South Africa. South African Journal of Child Health. 2015;9:36–7.

[10] Samb B, Desai N, Nishtar S, Mendis S, Bekedam H, Wright A, Prevention and management of chronic disease: a litmus test for health-systems strengthening in low-income and middle-income countries. The Lancet. 2010;376(9754):1785–97. 10.1016/S0140-6736(10)61353-0 21074253

[11] Lewandowski LB, Schanberg LE, Thielman N, Phuti A, Kalla AA, Okpechi I, Severe disease presentation and poor outcomes among pediatric systemic lupus erythematosus patients in South Africa. Lupus. 2016;26(2):186–94. 27488473 10.1177/0961203316660625 PMC5290292

[12] Harrison MJ, Zuhlke LJ, Lewandowski LB, Scott C. Pediatric systemic lupus erythematosus patients in South Africa have high prevalence and severity of cardiac and vascular manifestations. Pediatric rheumatology online journal. 2019;17(1):76-. 31771606 10.1186/s12969-019-0382-x PMC6878620

[13] Lewandowski LB, Watt MH, Schanberg LE, Thielman NM, Scott C. Missed opportunities for timely diagnosis of pediatric lupus in South Africa: a qualitative study. Pediatric Rheumatology. 2017;15(1):14. 28231857 10.1186/s12969-017-0144-6 PMC5322669

[14] Brunner HI, Huggins J, Klein-Gitelman MS. Pediatric SLE-towards a comprehensive management plan. Nature Reviews Rheumatology. 2011;7(4):225. 21386795 10.1038/nrrheum.2011.15

[15] Stats S. Statistics South Africa. Formal census. 2011.

[16] Guest G, MacQueen KM, Namey EE. Validity and reliability (credibility and dependability) in qualitative research and data analysis. Applied thematic analysis London: Sage Publications. 2012:79–106.

[17] Africa SS. Poverty trends in South Africa: An examination of absolute poverty between 2006 and 2011. Statistics South Africa Pretoria; 2014.

[18] Lehohla P Use of health facilities and levels of selected health conditions in South Africa: Findings from the General Household Survey, 2011: Statistics South Africa Pretoria; 2013.

[19] South Africa: South African Government; 2014 [Available from: https://www.gov.za/services/child-care-social-benefits/child-support-grant.

[20] Qu H, Shewchuk RM, Alarcon G, Fraenkel L, Leong A, Dall’Era M, Mapping Perceptions of Lupus Medication Decision-Making Facilitators: The Importance of Patient Context. Arthritis care & research. 2016;68(12):1787–94. 27059939 10.1002/acr.22904

[21] De Man J, Mayega RW, Sarkar N, Waweru E, Leys M, Van Olmen J, Patient-centered care and people-centered health systems in sub-Saharan Africa: Why so little of something so badly needed? 2016.

[22] Zhu TY, Tam LS, Li EK. Cost-of-illness studies in systemic lupus erythematosus: A systematic review. Arthritis care & research. 2011;63(5):751–60. 21557530 10.1002/acr.20410

[23] de-Graft Aikins A, Unwin N, Agyemang C, Allotey P Campbell C, Arhinful D. Tackling Africa’s chronic disease burden: from the local to the global. Global Health. 2010;6:5. 20403167 10.1186/1744-8603-6-5 PMC2873934

[24] Coleman K, Mattke S, Perrault PJ, Wagner EH. Untangling Practice Redesign from Disease Management: How Do We Best Care for the Chronically Ill? Annual Review of Public Health. 2009;30(1):385–408. 10.1146/annurev.publhealth.031308.100249 18925872

[25] Stellefson M, Dipnarine K, Stopka C. Peer reviewed: The chronic care model and diabetes management in US primary care settings: A systematic review. Preventing chronic disease. 2013;10. 10.5888/pcd10.120180 PMC3604796 23428085

[26] Ronksley PE, Sanmartin C, Campbell DJT, Weaver RG, Allan GM, McBrien KA, Perceived barriers to primary care among western Canadians with chronic conditions. Health Reports. 2014;25:3+. 24744042

[27] Busse R, Organization WH, Blumel M, Systems EOoH, Policies. Tackling Chronic Disease in Europe: Strategies, Interventions and Challenges: World Health Organization; 2010.

[28] Lewandowski LB. Tackling global challenges in pediatric rheumatology. Current opinion in rheumatology. 2020;32(5):414–20. 32657804 10.1097/BOR.0000000000000726

[29] Bisson GP Stringer JSA. Lost but Not Forgotten-The Economics of Improving Patient Retention in AIDS Treatment Programs. PLOS Medicine. 2009;6(10):e1000174. 19859529 10.1371/journal.pmed.1000174 PMC2760758

[30] Rachlis B, Naanyu V, Wachira J, Genberg B, Koech B, Kamene R, Identifying common barriers and facilitators to linkage and retention in chronic disease care in western Kenya. BMC Public Health. 2016;16(1):741. 27503191 10.1186/s12889-016-3462-6 PMC4977618

[31] Magadzire BP Mathole T, Ward K. Reasons for missed appointments linked to a public-sector intervention targeting patients with stable chronic conditions in South Africa: results from in-depth interviews and a retrospective review of medical records. BMC family practice. 2017;18(1):1–10. 28836941 10.1186/s12875-017-0655-8 PMC5571491

[32] Bailey ZD, Krieger N, Agenor M, Graves J, Linos N, Bassett MT. Structural racism and health inequities in the USA: evidence and interventions. The Lancet. 2017;389(10077):1453–63. 10.1016/S0140-6736(17)30569-X 28402827

[33] Paradies Y, Ben J, Denson N, Elias A, Priest N, Pieterse A, Racism as a determinant of health: a systematic review and meta-analysis. PloS one. 2015;10(9):e0138511. 26398658 10.1371/journal.pone.0138511 PMC4580597

[34] Norman R, Matzopoulos R, Groenewald P Bradshaw D. The high burden of injuries in South Africa/Forte charge de morbidite due aux traumatismes en Afrique du Sud/La gran carga de lesiones en Sudafrica. Bulletin of the World Health Organization. 2007 2007/09//:695+. 18026626 10.2471/BLT.06.037184 PMC2636399

[35] Mendenhall E, Norris SA. Diabetes care among urban women in Soweto, South Africa: a qualitative study. BMC Public Health. 2015;15(1):1300. 26706228 10.1186/s12889-015-2615-3 PMC4691296

[36] Peltzer K, Matseke G, Mzolo T, Majaja M. Determinants of knowledge of HIV status in South Africa: results from a population-based HIV survey. BMC Public Health. 2009;9(1):174. 19500373 10.1186/1471-2458-9-174 PMC2700104

[37] Tsang-A-Sjoe MW, Bultink IE, Heslinga M, Voskuyl AE. Both prolonged remission and Lupus Low Disease Activity State are associated with reduced damage accrual in systemic lupus erythematosus. Rheumatology. 2016:kew377. 10.1093/rheumatology/kew377 27803306

[38] Migowa A, Bernatsky S, Ngugi AK, Foster HE, Muriuki P Riang’a RM, Bridging gaps: a qualitative inquiry on improving paediatric rheumatology care among healthcare workers in Kenya. Pediatric Rheumatology. 2023;21(1):144. 38093255 10.1186/s12969-023-00935-3 PMC10717234

[39] Smith EM, Ainsworth S, Beresford MW, Buys V, Costello W, Egert Y, Establishing an international awareness day for paediatric rheumatic diseases: reflections from the inaugural World Young Rheumatic Diseases (WORD) Day 2019. Pediatric Rheumatology. 2020;18(1):1–6. 32917217 10.1186/s12969-020-00465-2 PMC7488434

[40] Gilbert L, Walker L. ‘My biggest fear was that people would reject me once they knew my status.’: stigma as experienced by patients in an HIV/AIDS clinic in Johannesburg, South Africa. Health & Social Care in the Community. 2010;18(2):139–46. 19708868 10.1111/j.1365-2524.2009.00881.x

[41] Campbell C, Foulis CA, Maimane S, Sibiya Z. “I Have an Evil Child at My House”: Stigma and HIV/AIDS Management in a South African Community. American Journal of Public Health. 2005;95(5):808–15. 15855456 10.2105/AJPH.2003.037499 PMC1449259

[42] Aggarwal A, Srivastava P Childhood onset systemic lupus erythematosus: how is it different from adult SLE? Int J Rheum Dis. 2015;18(2):182–91. 24965742 10.1111/1756-185X.12419

[43] Brennan KA, Creaven A-M. Living with invisible illness: social support experiences of individuals with systemic lupus erythematosus. Quality of life research. 2016;25(5):1227–35. 26449351 10.1007/s11136-015-1151-z

[44] Joachim G, Acorn S. Stigma of visible and invisible chronic conditions. Journal of Advanced Nursing. 2000;32(1):243–8. 10886457 10.1046/j.1365-2648.2000.01466.x

[45] McGrady ME, Hommel KA. Medication Adherence and Health Care Utilization in Pediatric Chronic Illness: A Systematic Review. Pediatrics. 2013;132(4):730. 23999953 10.1542/peds.2013-1451 PMC3784296

[46] Knight A, Weiss P Morales K, Gerdes M, Rearson M, Vickery M, Identifying Differences in Risk Factors for Depression and Anxiety in Pediatric Chronic Disease: A Matched Cross-Sectional Study of Youth with Lupus/Mixed Connective Tissue Disease and Their Peers with Diabetes. The Journal of Pediatrics. 2015;167(6):1397–403.e1. 26316371 10.1016/j.jpeds.2015.07.048 PMC5289225

[47] Chang JC, Davis AM, Klein-Gitelman MS, Cidav Z, Mandell DS, Knight AM. Impact of Psychiatric Diagnosis and Treatment on Medication Adherence in Youth With Systemic Lupus Erythematosus. Arthritis care & research. 2021;73(1):30–8. 32937032 10.1002/acr.24450

[48] Ku GMV, Kegels G. Adapting chronic care models for diabetes care delivery in low-and-middle-income countries: A review. World J Diabetes. 2015;6(4):566–75. 25987954 10.4239/wjd.v6.i4.566 PMC4434077

[49] Feldman CH, Bermas BL, Zibit M, Fraser P Todd DJ, Fortin PR, Designing an intervention for women with systemic lupus erythematosus from medically underserved areas to improve care: a qualitative study. Lupus. 2013;22(1):52–62. 23087258 10.1177/0961203312463979 PMC3543784

[50] Migowa A, Bernatsky S, Ngugi A, Foster HE, Muriuki P Lusambili A, An iceberg I can’t handle: a qualitative inquiry on perceptions towards paediatric rheumatology among healthcare workers in Kenya. Pediatric Rheumatology. 2023;21(1):6. 36681840 10.1186/s12969-023-00790-2 PMC9862847

[51] Makhlouf Y, Nessib DB, Ferjani H, Triki W, Maatallah K, Dhia K. The concept of telemedicine in pediatric rheumatology in Tunisia: Parents’ perceptions. Journal of Pediatric Nursing. 2023;69:6–9. 36584592 10.1016/j.pedn.2022.12.005 PMC9797351

[52] Migowa AN, Hadef D, Hamdi W, Mwizerwa O, Ngandeu M, Taha Y, Pediatric rheumatology in Africa: thriving amidst challenges. Pediatric Rheumatology. 2021;19(1):69. 33962643 10.1186/s12969-021-00557-7 PMC8103667

[53] Lewandowski LB, Hsieh E. Global rheumatology in the time of COVID-19. The Lancet Rheumatology. 2020;2(5):e254–e5. 32368736 10.1016/S2665-9913(20)30091-6 PMC7193143

[54] Shenoi S, Hayward K, Curran ML, Kessler E, Mehta JJ, Riebschleger MP Telemedicine in pediatric rheumatology: this is the time for the community to embrace a new way of clinical practice. Pediatric Rheumatology. 2020;18(1):1–4. 33129319 10.1186/s12969-020-00476-z PMC7602754

[55] Barak A, Boniel-Nissim M, Suler J. Fostering empowerment in online support groups. Computers in Human Behavior. 2008;24(5):1867–83.

[56] Al-Mutairi KD, Al-Zahrani MS, Bahlas SM, Kayal RA, Zawawi KH. Periodontal findings in systemic lupus erythematosus patients and healthy controls. Saudi medical journal. 2015;36(4):463. 25828284 10.15537/smj.2015.4.10746 PMC4404481

